# Ankle voluntary movement enhancement following robotic-assisted locomotor training in spinal cord injury

**DOI:** 10.1186/1743-0003-11-46

**Published:** 2014-03-31

**Authors:** Deborah Varoqui, Xun Niu, Mehdi M Mirbagheri

**Affiliations:** 1 Sensory Motor Performance Program, Rehabilitation Institute of Chicago, Chicago, USA; 2 Department of Physical Medicine and Rehabilitation, Northwestern University, Chicago, USA

## Abstract

**Background:**

In incomplete spinal cord injury (iSCI), sensorimotor impairments result in severe limitations to ambulation. To improve walking capacity, physical therapies using robotic-assisted locomotor devices, such as the Lokomat, have been developed. Following locomotor training, an improvement in gait capabilities—characterized by increases in the over-ground walking speed and endurance—is generally observed in patients. To better understand the mechanisms underlying these improvements, we studied the effects of Lokomat training on impaired ankle voluntary movement, known to be an important limiting factor in gait for iSCI patients.

**Methods:**

Fifteen chronic iSCI subjects performed twelve 1-hour sessions of Lokomat training over the course of a month. The voluntary movement was qualified by measuring active range of motion, maximal velocity peak and trajectory smoothness for the spastic ankle during a movement from full plantar-flexion (PF) to full dorsi-flexion (DF) at the patient’s maximum speed. Dorsi- and plantar-flexor muscle strength was quantified by isometric maximal voluntary contraction (MVC). Clinical assessments were also performed using the Timed Up and Go (TUG), the 10-meter walk (10MWT) and the 6-minute walk (6MWT) tests. All evaluations were performed both before and after the training and were compared to a control group of fifteen iSCI patients.

**Results:**

After the Lokomat training, the active range of motion, the maximal velocity, and the movement smoothness were significantly improved in the voluntary movement. Patients also exhibited an improvement in the MVC for their ankle dorsi- and plantar-flexor muscles. In terms of functional activity, we observed an enhancement in the mobility (TUG) and the over-ground gait velocity (10MWT) with training. Correlation tests indicated a significant relationship between ankle voluntary movement performance and the walking clinical assessments.

**Conclusions:**

The improvements of the kinematic and kinetic parameters of the ankle voluntary movement, and their correlation with the functional assessments, support the therapeutic effect of robotic-assisted locomotor training on motor impairment in chronic iSCI.

## Background

Individuals suffering from neurological diseases such as spinal cord injury (SCI) reveal several sensorimotor impairments. Previous studies report loss of voluntary control [1], muscle weakness, i.e., an inability to generate normal levels of muscle force [2,3], abnormal increase in muscle tone, i.e., spasticity [2,4], and abnormal co-activation in muscular patterns [5,6]. A majority of patients with incomplete motor SCI (iSCI) recovers a certain level of walking capacity but presents severe limitations [7]. Gait in iSCI is characterized by a number of features, including reduced speed, abnormal kinematic patterns and disrupted muscle activity [8]. To improve over-ground walking ability, locomotor therapies that combine a body-weight support (BWS) system with a treadmill have been developed over the past two decades [9,10]. BWS training is sometimes coupled with manual assistance from physical therapists [11,12], functional electrical stimulation [13,14], or most recently with robotic assistance [15,16]. Currently, there is no consensus regarding the most beneficial therapy to enhance ambulation in iSCI patients [17,18]. Robotic-assisted locomotor present several advantages, including the ability to increase the intensity and the total duration of training while maintaining a physiological gait pattern. Also, the task-specific practice of stepping is known to enhance the afferent feedback associated with normal locomotion and can induce plasticity in the involved motor centers [19-22]. Moreover, locomotor robotic devices can reduce personnel costs involved in manual assistance training (which can require up to three physical therapists) and can prevent excessive fatigue induced by electrical stimulation.

The Lokomat® (Hocoma AG, Volketswill, Switzerland), a driven-gait orthosis (DGO) device attached to a treadmill with BWS, was developed for locomotor therapy for various neurological diseases [23]. The device is composed of a motorized exoskeleton with bilateral electric drives that deliver a guidance force to the hip and knee joints, providing assistance during the swing and stance phases of the gait cycle. Previous research has shown that Lokomat training helps iSCI patients to improve their over-ground speed and gait endurance [15,16,24,25]. However, the mechanisms underlying these improvements are still unclear. The effectiveness of the robotic-assisted locomotor therapies is mostly quantified using clinical gait outcome measures (e.g., walking speed and functional independence); too few studies have investigated its specific effects on structures and functions involved in locomotion (e.g., neuroplasticity, muscle strength, gait pattern, etc.) [22,26-28]. However, recent work focused on understanding the mechanisms by which Lokomat training improves walking recovery capabilities seem promising. For example, it appears that training using the robotic device increases lower limbs’ muscular activity [28,29] or cerebellum activation [22]. Moreover, Hidler et al. [26] demonstrated that, in a healthy population, the range of motion at the ankle joint is larger inside the Lokomat than on a treadmill. A larger range of motion could be helpful to improve the foot clearance during the swing phase and reduce the foot drop syndrome in iSCI.

The foot drop syndrome is considered as a strong limiting factor in the restoration of locomotion for those with neurological disease [8,30]. It is associated with a poor quality of locomotion, reduced functional capacity [31], and an enhanced risk of falling [32]. Researchers have observed deficient propulsion at the end of the stance phase, i.e., low plantar-flexion (PF) moment [33,34], and foot drag during the swing phase, i.e., reduced dorsi-flexion (DF) movement [33,35]. Dorsi-flexors— particularly the tibialis anterior (TA) — play an important role in controlling the foot trajectory during the swing phase to ensure adequate foot clearance phase and to prevent foot drag. TA weakness can lead to reduce range of motion and reduced velocity peak during voluntary DF [36,37]. In iSCI patients, muscle weakness results from the combination of a deficit in cortico-spinal voluntary activation [38,39], muscular atrophy [40,41] and increased stiffness in antagonist muscles [42,43]. Thus, locomotor training using the Lokomat seems promising to reduce these deficits and improve ankle DF movement.

This study focused on changes in ankle voluntary motor control in iSCI patients, considered as a strong indicator of recovery [44], after a robotic-assisted locomotor therapy. The aim was to quantify the effects of a 4-week Lokomat training regimen on the impaired voluntary movement of the ankle by measuring kinetic and kinematic parameters during a DF movement, and on patients’ walking capacities assessed with popular clinical scales.

## Method

### Participants

Thirty ambulatory chronic SCI subjects with incomplete motor function loss due to trauma were recruited to participate in this study and randomly assigned to the two study groups. Half of the participants performed a 1-month Lokomat training (i.e., Intervention group) and the other half was assigned to the Control group. Groups were matched by age, time since injury, muscle tone at the ankle plantar-flexors assessed with the 6-point ordinal Modified Ashworth Scale (MAS) [45,46], and walking ability measured with the Walking Index for Spinal Cord Injury II (WISCI II). WISCI II assesses the patient’s need for assistance or helping aids while walking over a short distance, using a scale ranging from 0 (patient unable to stand and/or participate in assisted walking) to 20 (patient ambulates with no device or physical assistance) [47,48]. Demographic characteristics for both groups are presented in Table 1.

**Table 1:** Baseline demographic characteristics and clinical scores of the Lokomat and Control groups

	**Lokomat group (n = 15)**		**Control group (n = 15)**	
	**Mean ± S.D.**	**Range**	**Mean ± S.D.**	**Range**
**Age (y)**	50.80 ± 2.12	37–70	44.65 ± 2.66	25–62
**Lesion duration (y)**	11.80 ± 2.54	2–36	8.09 ± 1.89	1–27
**WISCI II (range, 0–20)**	15.33 ± 1.12	9–20	14.20 ± 1.47	9–20
**TUG (s)**	34.2 ± 9.6	9.7–126.1	39.7 ± 6.76	8.7–95.0
**10MWT (m/s)**	0.56 ± 0.09	0.05–1.06	0.56 ± 0.11	0.09–1.49
**6MWT (m)**	207.0 ± 29.6	25.5–346.0	205.6 ± 35.3	25.9–501.2
**Sex**^**† **^**(F - M)**	7 (46.67%) - 8 (53.33%)	1 (6.67%) - 14 (93.33%)		
**Level of lesion**^**† **^**(C [C2-C7] – T [T1-T7])**	11 (60) - 4 (40)	9 (73.33) - 6 (26.67)		
**Ankle flexors MAS**^**‡ **^**(range, 0–4)**	2 (IQR = 1)	2 (IQR = 1)		

Continuous data are expressed as Mean ± Standard Deviation [range], with the exception of categorical variables (^†^) expressed as a number (%) and nominal variables (^‡^) expressed as the median (InterQuartile Range). *Abbreviations*: *F*, Female; *M*, Male; *C*, Cervical; *T*, Thoracic; *MAS*, Modified Ashworth Scale; *WISCI II*, Walking Index for Spinal Cord Injury II; *TUG*, Timed Up and Go; *10MWT*, 10-meter walk; *6MWT*, 6-minute walk.

All the participants met the following inclusion criteria: (a) motor-incomplete SCI with an American Spinal Injury Association impairment scale classification of C or D [49], (b) level of injury above T10, (c) ambulatory (i.e., showing the ability to take at least one step independently), (d) passive range of motion of the leg joints within functional limits for ambulation, and (e) medical clearance to participate. Patients from both groups were instructed to maintain a constant level of activity over the duration of the experiment. Subjects were draw from the outpatient service of the Rehabilitation Institute of Chicago. All of them provided their informed consent and the Northwestern University Institutional Review Board approved the study.

### Locomotor training

Participants from the Intervention group participated in robot-assisted locomotor training using the Lokomat three times a week over four weeks, for a total of twelve training sessions. Each session lasted one hour, including set-up time, with between 30 and 45 minutes of training. The treadmill speed was increased from 1.5 km/h to 3.0 km/h, as determined by patient tolerance and comfort, over the course of training. The guidance force, i.e., the level of control of the hip and knee joint trajectories in the sagittal plane provided by DGO, was progressively reduced during the training (from full to 20% assistance), as tolerated by the patient. Participants were instructed to “walk with the robot” to ensure lower-extremity movements that were consistent with the Lokomat stepping pattern. During the training, reductions in guidance force were prioritized over increasing walking speed to minimize the passive locomotor training and promote maximal voluntary muscle activation. The body-weight support was configured (from 95% to 25% body weight) to maximize lower-extremity loading without producing excessive knee flexion during the stance phase or allowing to toes to drag during the swing phase. The three training parameters (i.e., walking speed, guidance force and body-weight support) were adjusted to guarantee the highest amount of active subject participation and to provide a challenge to them over the training’s sessions. Straps secured the heads of the metatarsals to the foot supports on the robot to ensure a neutral position of the ankle (i.e., ankle angle of 90°). Patients were asked to pay attention to their ankle movements during the gait cycle. We encouraged patients to lift their toes during the swing phase and focus on planting the heel of the forward foot on the treadmill first. A mirror was placed before the patient to allow him to monitor his leg movements during the training.

### Clinical assessments

Patients performed three clinical evaluations to assess their functional ambulation capacity, including the Timed Up and Go test (TUG), the 10-meter walk test (10MWT), and the 6-minute walk test (6MWT). TUG evaluates the mobility and functional ambulation by measuring time taken by the subject to stand up from an armed chair, walk for 3 meters, turn around, return, and sit down into the chair [50]. 10MWT is used to evaluate preferred walking speed by measuring the time spent to walk a distance of 10 meters [51].

6MWT is used to assess walking endurance capacity by measuring the distance in meters covered by 6 minutes of walking [31]. Clinical evaluations were performed at two time intervals: before (baseline) and after the complete Lokomat training period (one month) for the Intervention group; for the Control group, baseline and 1-month evaluations were also performed. Baseline scores for both groups are presented in Table 1.

### Ankle kinematic and kinetic assessments

#### Apparatus and recording

**Figure 1:**
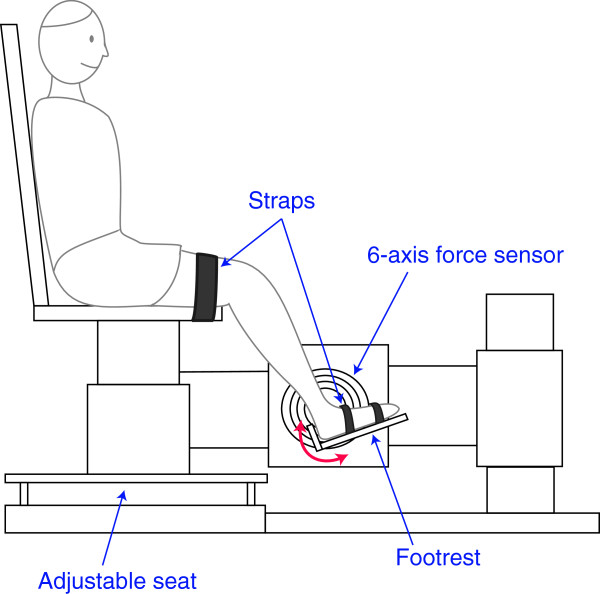
**Experimental setup.** Experimental apparatus used to evaluate ankle voluntary dorsi-flexion movement and isometric maximal voluntary contractions.

The voluntary movement capacity and muscle strength of the ankle were evaluated at the same two time intervals as the clinical assessments (i.e., baseline and 1-month). For this purpose, participants were seated in an experimental chair with the thigh strapped to the chair base (Figure 1). The ankle was secured to a rigid “footrest” which was in turn attached to the rotational axis of a servomotor, allowing for DF and PF of the ankle. The seat and footrest were adjusted such that the ankle and knee joints were flexed at 90° (ankle) and 130° (knee). A rotary encoder recorded angular position, while a 6-axis torque transducer recorded torque; this transducer was aligned such that the torque was measured about the ankle axis of rotation. Position and torque data were sampled at 1 kHz by a 16 bit A/D converter, and anti-alias filtered on-line at 200 Hz. A 90° angle between the calf and foot was considered as the neutral position for the ankle and defined as zero degrees; DF was considered positive by convention.

#### Experimental procedure

To evaluate DF voluntary movement capacity, subjects were instructed to rotate their ankle from full PF position to full DF position at their maximum possible speed. The initial full PF position was defined as the maximal position voluntarily reached by the subject in the PF direction without assistance. We repeated this task twice.

Prior to the voluntary task, the passive range of motion (PROM) was determined by an examiner manually stretching the patient’s ankle attached to the footrest in both directions, at a very slow (quasi-static) speed as to prevent muscle activation, until maximal resistance or pain was encountered.

We also measured the isometric maximal voluntary contraction (MVC) of the ankle plantar- and dorsi-flexors while the ankle was held fixed at the neutral position. Participants were instructed to voluntarily contract their plantar-flexors by pushing down with their toes onto the footrest with as much effort as possible; to contract dorsi-flexors, patients were instructed to lift their toes up. Subjects were encouraged to sustain the MVC contraction for 5 seconds in each direction.

While SCI typically affects both sides of the body, in this study only the ankle with the lowest isometric maximum voluntary torque in the DF direction and the highest MAS score was examined.

#### Data reduction

**Figure 2:**
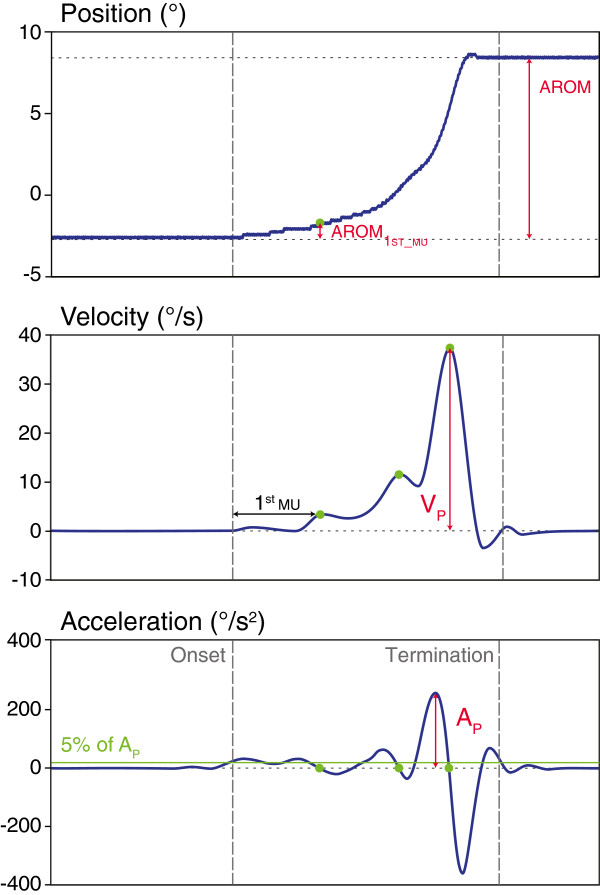
**Movement trajectories at the ankle joint in voluntary dorsi-flexion.** Movement trajectories of ankle angular position **(top)**, velocity **(middle)** and acceleration **(bottom)** of a typical participant at baseline during the voluntary movement task from full plantar-flexion (PF) to full dorsi-flexion (DF). AROM = full active range of motion; 1stMU = First movement unit; AROM_1stMU_ = AROM between the onset and 1stMU; %AROM_1stMU_ = percentage of AROM_1stMU_ compared to full AROM; V_P_ = maximum velocity peak; A_P_ = maximum acceleration peak.

Angular velocity and acceleration were computed as the first and second derivatives of the ankle angular position (Figure 2). The onset of movement was defined as the first sample whose acceleration was greater than 5% of the maximum acceleration peak; the termination of movement was defined once the acceleration dropped below this value. The first movement unit (1stMU) was defined as the first peak (local maximum) in velocity after the movement onset. The times of peak velocity were detected in the acceleration profile as the time points where the acceleration changed sign from positive (acceleration) to negative (deceleration) through zero.

From this data, the full active range of motion (AROM) at the ankle joint was computed between the onset and the termination of movement, as well as the maximum angular velocity peak (V_P_). Smoothness of the movement was estimated using two variables: (i) AROM_1stMU_, the AROM between the onset and the first movement unit, and (ii) %AROM_1stMU_, the percentage of AROM_1stMU_ compared to the full AROM. Our earlier study suggests that these two variables are sensitive enough to detect changes in movement smoothness in SCI patients [52]. These kinematic measures indicate the ability of the patient to scale muscle force to accomplish the movement in a single accelerating and decelerating cycle [52].

Maximum isometric torque was computed during the 5-second MVC hold in each direction (DF and PF). The average torque during the 3-second window in which the torque exhibited the smallest standard deviation was used as the MVC torque; the largest magnitude plantar-flexion (MVC_PF_) and dorsi-flexion (MVC_DF_) torque of the two trials was used.

#### Statistical analysis

Since Kolmogorov-Smirnov tests of normality indicated a skewed distribution of data, non-parametric tests were used for all the following analysis.

To test the homogeneity between Control and Intervention groups at the inclusion, Wilcoxon signed-rank tests were conducted on the continuous variables (i.e., Age, Lesion duration and WISCI II) and Chi-squared tests were performed on the categorical and ordinal variables (i.e., Level of lesion and MAS).

In order to determine the effects of the Lokomat training on the ankle voluntary movement and walking capacities, we compared the MVC values, the kinematic parameters for the DF task, and the clinical evaluations of walking capacity assessed at baseline and one month. Wilcoxon signed-rank tests were performed on baseline and 1-month data independently for each group for the following parameters: PROM, AROM, V_P_, AROM_1stMU_, %AROM_1stMU_, MVC_PF_, MVC_DF_, TUG, 10MWT and 6MWT.

We used Spearman rank correlation analysis to determine the relationships between the three clinical assessments (i.e., TUG, 10MWT and 6MWT) and the ankle’s kinematic and kinetic parameters at baseline (i.e., AROM, V_P_, AROM_1stMU_, %AROM_1stMU_, MVC_PF_ and MVC_DF_ ).

The significance level was set at P = 0.05.

## Results

### Relationship between walking assessments and ankle ability measures

We investigated the relationship between ankle voluntary movement capabilities (quantified by our kinematic and kinetic variables) and walking capacities (assessed by clinical scales) using correlation analysis on baseline measurements. The correlation coefficients are presented in Table 2. Both the mobility assessed with the TUG and the over-ground walking speed measured with the 10MWT showed significant correlations with AROM, AROM_1stMU_, MVC_PF_ and MVC_DF_ (All Ps < 0.05). Thus, these two components of ambulation are related to ankle ability, as measured by active range of motion, movement smoothness and muscle strength. Moreover, the 6MWT assessing the endurance capacity was significantly correlated with AROM, V_P_, AROM_1stMU_ and MVC_DF_ (All Ps < 0.05). To correct for these multiple tests, we have used Bonferroni correction to adjust the alpha level for all our correlation analysis. Since we made 18 tests, a level of significance was set at 0.0028 (0.05/18) for which all correlations reported significant (Table 2) remained so except one related to the relationship between TUG and MVC_PF_.

**Table 2:** Correlations (r values) between the walking clinical assessments and the ankle voluntary movement parameters at baseline

	**Kinematic**				**Kinetic**	
	**AROM**	**V**_**P**_	**AROM**_**1stMU**_	**%AROM**_**1stMU**_	**MVC**_**PF**_	**MVC**_**DF**_
**TUG**	−0.57*	−0.37	−0.47*	0.22	−0.41*	−0.52*
**10MWT**	−0.53*	−0.34	−0.48*	−0.11	−0.44*	−0.50*
**6MWT**	0.70*	0.73*	0.74*	0.02	0.36	0.63*

*Abbreviations*: *TUG*, Timed Up and Go; *10MWT*, 10-meter walk test; *6MWT*, 6-minute walk test, *AROM*, Active Range of motion; *V*_*P*_, Velocity peak. *AROM*_*1stMU*_, AROM between the onset and the first movement unit; %*AROM*_*1stMU*_, Percentage of AROM_1stMU_ compared to full AROM; *MVC*_*PF*_*and MVC*_*DF*_, Maximal voluntary contraction in dorsi- and plantar-flexion directions. *represents significant correlations (P < 0.05).

To summarize, correlation tests show that the variables used to describe the kinematic components of the DF voluntary movement and the muscle strength at the ankle joint were related to popular measures of mobility and walking capacities used in the clinic.

### Therapeutic effects of robotic-assisted locomotor training

At the first evaluation (baseline), the two groups were homogeneous. No statistically significant differences were found in terms of age (P = 0.09), level of lesion (χ2 = 0.6, df = 1, P = 0.44), duration of lesion (P = 0.46), walking capacity measured with WISCI II (P = 0.61) and plantar-flexor muscle tone assessed with MAS (χ2 = 5.12, df = 4, P = 0.27).

#### Ankle passive range of motion and muscle tone

For the Intervention group, a significant improvement in the ankle PROM was observed over time (P < 0.05). The mean ± standard error of PROM was 62.33° ± 2.06° at baseline and 64.33° ± 2.23° at one month. For the Control group, PROM was similar between the two evaluations (baseline: 58.00° ± 2.62°; 1-month: 57.33° ± 2.92°; P = 0.75).

Regarding plantar-flexor muscle tone assessed with MAS, no change is observed in the 1-month interval for both groups (Intervention group: χ2 = 5.78, df = 4, P = 0.22; Control group: χ2 = 4.50, df = 4, P = 0.21).

#### Ankle dorsiflexion voluntary movement: kinematic measures

**Figure 3:**
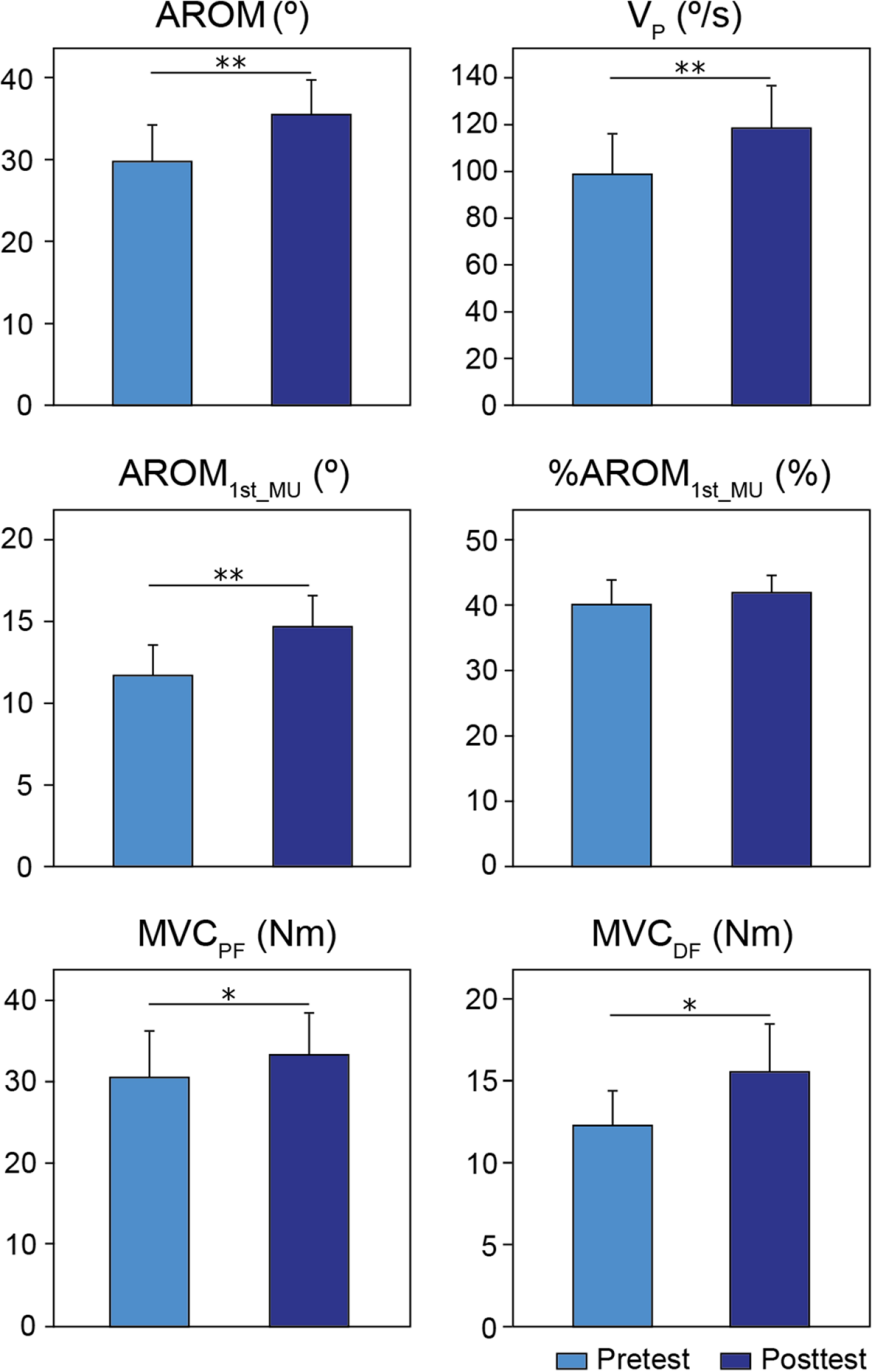
**Baseline and 1-month average in the ankle voluntary movement for the Intervention group.** Kinematic (AROM and V_P_), smoothness (AROM_1stMU_ and %AROM_1stMU_) and kinetic (MVC_PF_ and MVC_DF_) group averages before and after one month of Lokomat training. Bars represent the standard error of the group mean. Asterisks indicate a statistical comparison between pre- and posttests, where *indicates significance at P < 0.05; **indicates significance at P < 0.01.

For the Intervention group, the analysis showed that three of the four kinematic parameters that were used to quantify the voluntary DF performance presented an improvement over the course of the Lokomat training. Paired-comparisons revealed a significantly greater AROM (P < 0.05), V_P_ (P < 0.05) and AROM_1stMU_ (P < 0.05) at one month compared to baseline (Figure 3). For AROM, subjects exhibited an average increase of 27.5 ± 9.9% (5.13 ± 1.63°), improving from 28.80 ± 3.84° to 33.93 ± 3.89°. For V_P_, we observed an average increase of 27.54 ± 12.24% (15.90 ± 7.16°/s); at baseline the average maximum velocity peak was 93.97 ± 13.49°/s, while it had increased to 109.87 ± 14.54°/s after one month. For AROM_1stMU_, the average increase is of 38.88 ± 17.66% (2.79 ± 0.95°) from 11.48 ± 1.50° to 14.27 ± 1.79°. However, there is no significant change over time for %AROM_1stMU_ (P = 0.64). That is, patients from the Intervention group were able to voluntarily move their ankles farther and faster after training. In general, these participants exhibited an improvement in the kinematic and smoothness components of their movement during the voluntary DF task.

In contrast, for the Control group, no statistically significant difference was found between the two tests in AROM (P = 0.95), V_P_ (P = 0.34), AROM_1stMU_ (P = 0.59) and %AROM_1stMU_ (P = 0.50).

#### Ankle maximum isometric voluntary contractions

**Figure 4:**
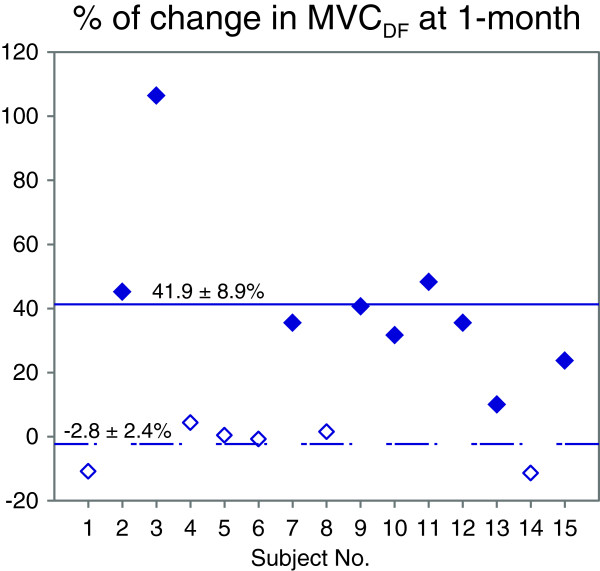
**Individual changes in dorsi-flexor muscle strength after training.** Percentage increase in the dorsi-flexion maximal voluntary contraction (MVC_DF_) at the posttest normalized by the pretest value for each subject. Subjects showing an improvement in the MVC_DF_ are represented by black symbols. White symbols represent the subjects experiencing no change over time.

We quantified the changes in plantar- and dorsi-flexor muscle strength at the ankle joint over the training, using baseline vs. 1-month comparisons of the mean MVC torque. For the Intervention group (Figure 3), an average increase of 14.79 ± 4.71% was observed in the PF direction (P < 0.05), with the mean torque increasing from 26.77 ± 4.42 Nm at baseline to 29.13 ± 4.10 Nm at one month. In the DF direction, MVC_DF_ improved significantly by 24.04 ± 7.91% at one month compared to baseline (P < 0.05), from a baseline mean of 10.95 ± 1.67 Nm to a one month mean of 13.55 ± 2.31 Nm. The relatively larger variance after training, compared to baseline, in the DF direction suggests high variability in individual responses to the Lokomat training as illustrated in Figure 4. We observed two different recovery trends: nine of our fifteen participants (63% of the subjects) presented an increase in MVC_DF_ of 10-106% (average change: 41.9 ± 8.9%), while the rest of the group experiences no change over time (average change: −2.8 ± 2.4%).

In summary, results of MVC tests show an improvement in the strength of dorsi- and plantar-flexor muscles during isometric voluntary contraction after the 1-month Lokomat training. On the other hand, for the Control group, no change was observed between the two tests in MVC_PF_ (P = 0.09) and MVC_DF_ (P = 0.81).

#### Walking performance

To evaluate the effectiveness of the Lokomat training in terms of functional capabilities, we compared the performance of the Intervention group for three clinical measures of ambulation impairment (i.e., TUG, 10MWT and 6MWT) before and after the training. The TUG showed a significant reduction of 13.99 ± 3.53% in the time needed to perform the task (P < 0.05). The mean time to perform the task was 34.15 ± 9.61 s before training and 27.83 ± 7.32 s after the Lokomat training. The 10MWT exhibited a significant increase in the mean over-ground gait speed (0.08 ± 0.02 m/s) after training (P < 0.05), from a mean speed of 0.56 ± 0.09 m/s pre-training to a mean speed of 0.64 ± 0.10 m/s post-training. This change corresponded to an improvement of 13.4 ± 2.8%. These results demonstrate an improvement in mobility (TUG) and walking speed (10MWT) after one month of robotic-assisted locomotor practice. However, no significant change in the patients’ endurance capacity was observed between the two tests, as assessed by the 6MWT (P = 0.46): the mean walking distance was 206.96 ± 29.57 m before training and 208.87 ± 28.36 m after the training.

For the Control group, no statistically differences were found between the two tests in TUG (P = 0.45), 10MWT (P = 0.36) and 6MWT (P = 0.17).

## Discussion

The present study is, to our knowledge, the first to have investigated the effects of a one-month Lokomat training program on the voluntary movement of the ankle joint in chronic iSCI patients. Our results demonstrated that a benefit of this locomotor training could be to improve ankle voluntary DF movement in terms of range of motion, velocity and smoothness. Moreover, a gain in the strength of the dorsi- and plantar-flexor muscles was seen after the Lokomat training. The clinical gait assessments performed indicated that the Lokomat training could drive an improvement in mobility and over-ground walking velocity. In addition, correlations observed between our kinematic and kinetic variables and these clinical measures suggest that the parameters examined in this study may help to better understand the mechanisms which underlie improvements in walking capacity following robotic-assisted locomotor training.

### Lokomat training effects on ankle muscle strength

To our knowledge, this is the first time that the effect of Lokomat training on the strength of the ankle plantar-flexor muscles was directly investigated. The improvement in plantar-flexor strength is a key element in gait recovery as the forward progression during locomotion is primarily produced by this muscle group [53,54]. Our results show that the maximal torque generated during the MVC_PF_ task increases significantly from the baseline after the Lokomat training. This finding confirms earlier observations that repetitive locomotor training in chronic iSCI patients is beneficial to developing strength in the PF muscle group [55]. This improvement could be the expression of the muscle plasticity phenomenon resulting from the extra loading on the lower limb [56,57], as well as an enhancement of voluntary activation drive by the intensive repetition of an “unused” task [55,58].

Weakness in the dorsi-flexor muscles is one of the major causes of impairment in foot clearance during the swing phase (i.e., foot drop syndrome) [30]. Thus, recovery of the strength of ankle dorsi-flexor muscles is essential to prevent excessive plantar-flexion movement. Our results indicate an increase in MVC_DF_ generated after the training. Only one earlier study reports an augmentation of the tibialis anterior muscle activity in a Lokomat training when resistance is added during the swing phase [29]. Similar to plantar-flexor muscles, the increase in MVC_DF_ level could find its origin in the improvement of voluntary activation of ankle dorsi-flexor muscles [38,59]. Previously, an enhancement in the cortico-spinal tract function has been observed for the tibialis anterior following treadmill training in chronic SCI patients [60]. In our point of view, the unique configuration provided by the Lokomat can be beneficial to improve ankle voluntary activation. By actively guiding hip and knee joints through DGO in a physiological stepping pattern, a patient can focus his attention on his ankle movements during the gait cycle. Moreover, the strap placed around footwear maintaining the foot in a neutral position allows the dorsi-flexor muscles contract to their shortened length, a condition known to promote the best recovery of function as suggested by Ada et al. [61].

A diminution of spasticity in the antagonist (i.e., plantar-flexor) muscles would reduce its reciprocal inhibitory effects on the dorsi-flexors muscles, particularly the TA activation [42,62,63], and could also explain the increase in MVC_DF_. In our study, no significant difference has been observed in plantar-flexor muscle tone assessed with MAS between baseline and 1-month. However reliability and reproducibility of MAS is unclear [64], particularly regarding the assessment of spasticity in the lower extremities in the SCI population [65-67]. A recent study showing a reduction of the reflex and intrinsic components of the ankle stiffness in chronic iSCI subjects undergoing Lokomat training offers some support to this hypothesis [68]. Also, the increase in MVC_DF_ might be the expression of the enhancement of voluntary activity in the TA due to the reduction of the inhibitory dorsiflexor responses. On the other hand, the lack of improvement in some subjects could be the consequence of atrophic dorsi-flexor muscles, a common phenomenon in chronic SCI patients [40].

The different recovery patterns observed in MVC_DF_ (i.e., an increase observed in half of our study participants and no change for the other half) should be investigated further to better understand the mechanisms underlying the recovery of dorsi-flexor muscle activity following Lokomat training.

### Ankle dorsi-flexion kinematic improvement

Ankle dorsi-flexion restoration is fundamental in iSCI patients to prevent foot drag and improve walking capabilities [31]. The correlation tests performed in this study showed a relationship between the walking clinical assessments (i.e., TUG, 10MWT and 6MWT) and the variables used to quantify the ankle dorsi-flexion voluntary movement (i.e., AROM, V_P_, AROM_1stMU_). Patients who presented the highest walking capacities also showed the best performance for the ankle voluntary movement from full PF position to full DF position. Our results show that after four weeks of Lokomat training, subjects increase their range of motion, their maximum velocity, and the smoothness of their motion.

At the ankle joint, the AROM (measured from maximal PF to maximal DF) is limited by the interaction between (i) the force produced by the dorsi-flexor muscles, (ii) the passive resistance from the stretched structures and (iii) the spastic co-contraction in the antagonist muscles (i.e., plantar-flexors). As the increase in MVC_DF_ was observed in nine of our fifteen patients, the gain observed in AROM for all our patients cannot be entirely due to an enhancement in dorsi-flexor muscle strength. On the other hand, the significant improvement in the passive range of motion found after the 1-month training suggests a reduction in the resistance of the passive tissues. During the Lokomat training, the support provided by foot straps can prevent both an excessively shortened position of the plantar-flexor muscles as well as incapacitating contractures. Thus, this “stretching” of calf muscles could have a beneficial effect on the passive ankle joint stiffness [69] and by extension improve the ankle DF voluntary movement. The reduction of the spastic co-contraction in the antagonist muscles observed after a Lokomat training in an earlier study would also serve to increase the ankle AROM [68]. In addition, a diminution in the spasticity in our subjects would explain the improvement in other parameters of the ankle DF voluntary movement (i.e., velocity peak and smoothness); previous studies have observed some degree of causality between spasticity and the velocity and smoothness of voluntary movement [70-73]. In fact, Latash and Penn [72] demonstrated an improvement in the peak speed and smoothness of ankle movement when muscle spasticity was reduced by anti-spastic medication.

### Walking capacity improvement

In terms of functional capacities, the results obtained for the clinical assessments show an improvement of mobility (i.e., TUG) and over-ground walking speed (i.e., 10MWT) after one month of Lokomat training for our iSCI subject group. These observations are consistent with the literature on robotic-assisted gait devices showing a beneficial effect on the ambulation capacities [15,16,18,22]. The improvement in walking speed is particularly noteworthy; we observed an average increase of 0.08 m/s, which is greater than the minimally important difference of 0.05 m/s reported by Musselman after a BWS treadmill training [73]. Two factors evaluated in this study might contribute to the observed gain in mobility and velocity: (i) improvements in the strength of the ankle plantar-flexor muscle group, which provides most of the energy required for forward progression [53,54], and (ii) enhancements to the dorsi-flexion voluntary movement, which allows for adequate clearance during the swing phase [8].

The absence of changes in the 6MWT—which evaluates the endurance component of the gait capacity—might be explained by the duration of the training provided to our participants. In the study, we administered twelve 1-hour Lokomat training sessions over a month, but the endurance gain in over-ground gait has been observed for robotic-assisted locomotor training studies with training durations that are at least twice as long [15,44]. A longer and/or more intensive training regimen might be helpful to develop endurance capacity in the chronic iSCI population.

### Study limitations and implications

Our study characterized the impaired voluntary movement at the ankle joint using a specific task that involved only the joint by itself, and only articulated the joint through PF and DF. The confirmation of our findings during a functional task as walking (e.g., an assessment of the spatio-temporal trajectories of the ankle joint during the gait cycle) would be helpful to confirm the clinical significance of the physical intervention (Lokomat training). Comparison of gait kinematics within this population can be complicated by differences in self-selected speed and gait pattern, and the presence of braces, orthotics, and gait assistance device (such as walkers). The present experimental design attempts to control for these differences by isolating only the DF/PF motion. Also, technical and methodological limitations prevent the assessment of many ankle kinematic and kinetic variables during standard gait. However, the relationships found between our variables and the clinical measures indicate that the ankle voluntary movement limitations are related to walking capacity, thus suggesting a therapeutic benefit of the Lokomat training.

The observation of individual trends in the recovery of dorsi-flexor strength illustrates that Lokomat training induces different patterns of recovery among patients, as expected from any therapeutic intervention. Thus, to fully characterize the effects of the robotic-assisted locomotor therapy, we need to further investigate the different recovery patterns identified in the recovery of dorsi-flexor muscle strength. A systematic assessment of the spasticity phenomenon at the ankle joint, as well as the identification of skeletal muscle atrophy and assessment of cortico-spinal function, would be helpful to determine the underlying mechanisms involved in the ankle voluntary movement recovery. A complete understanding of these mechanisms is crucial for identifying patients for whom a robotic-assisted locomotor therapy would be the most beneficial (i.e., to achieve the best functional outcome), as well as to specify an appropriate training duration and intensity which would maximize positive results.

Finally, researchers who performed the tests were not blinded for group allocation because they needed to stay with the subjects during the training and experimental protocol.

### Clinical significance

Our current findings suggest that a 1-month Lokomat training regimen is beneficial to improve ankle voluntary movement in a chronic iSCI population. Even if the Lokomat training does not provide an active control of the ankle, its impact on this joint should not be neglected, as well as the instructions given to maximize the contribution of the ankle during the training. The correlation of these parameters with the walking measures is promising to assess the efficacy of robotic-driven locomotor training. The specific pathways by which ankle voluntary movement is enhanced remain to be investigated. However, an improvement of voluntary activation of dorsi-flexors muscles associated with a reduction of passive and neuromuscular components of joint stiffness might explain the changes observed following the 1-month training. The findings of this study help to improve the understanding of how the Lokomat training affects the recovery of walking capacities in patients with chronic iSCI.

## Competing interest

The authors declare that they have no competing interests.

## Authors’ contributions

DV and XN were involved in subject recruitment, data collection, and Lokomat training. DV performed the analysis and interpretation of data and wrote the manuscript. XN assisted in revising the manuscript. MMM designed the study, supervised data collection and analysis, and participated in interpreting and revising the manuscript. All authors read and approved the final manuscript.
